# Crystal structure of (2*E*,4*E*)-5-[bis­(2-hy­droxy­eth­yl)amino]-1-(4-chloro­phen­yl)-5-phenyl­penta-2,4-dien-1-one

**DOI:** 10.1107/S2056989015019568

**Published:** 2015-10-24

**Authors:** Alexander A. Golovanov, Anna V. Vologzhanina, Ivan S. Odin, Tat’yana P. Tret’yakova, Sergey V. Naumov

**Affiliations:** a Department of Chemistry, Chemical Processes and Techologies, Togliatti State University, 445667 Togliatti, Russian Federation; b Nesmeyanov Institute of Organoelement Compounds of the Russian Academy of Sciences, 119991 Moscow, Russian Federation; c The Laboratory of Functional Heterocyclic Compounds, Togliatti State University, 445667 Togliatti, Russian Federation

**Keywords:** crystal structure, dienes, enamines, hydrogen bonding, C—H⋯π inter­actions

## Abstract

In the title compound, C_21_H_22_ClNO_3_, the penta­diene unit is nearly planar [maximum deviation = 0.023 (1) Å], but the carbonyl O atom deviates significantly [by 0.304 (1) Å] from its mean plane, which is twisted with respect to the phenyl and chloro­benzene rings by 71.34 (13) and 46.40 (13)°, respectively. In the crystal, inversion-related molecules are linked by two pairs of O—H⋯O hydrogen bonds, forming chains propagating along [01-1], enclosing *R* ^2^_2_(16) and *R* ^2^_2_(22) ring motifs. The chains are linked *via* C—H⋯O hydrogen bonds and C—H⋯π inter­actions into a three-dimensional supra­molecular architecture.

## Related literature   

For crystal structures of 1-aryl-5-phenyl­penta-2,4-dien-1-ones, see: Kashino & Haisa (1980 ▸); Fischer *et al.* (2007*a* ▸,*b* ▸); Patil *et al.* (2007 ▸); Zhao *et al.* (2007 ▸); Silva *et al.* (2011 ▸); Vologzhanina *et al.* (2013 ▸); Golovanov *et al.* (2014 ▸). For non-linear optical properties of 1,5-di­aryl­pent-2,4-dien-1-ones, see: Singh & Miyata (1996 ▸). For the biological activity of related chalcones, see: Karaman *et al.* (2012 ▸); Nielsen *et al.* (2005 ▸); Wu *et al.* (2011 ▸).
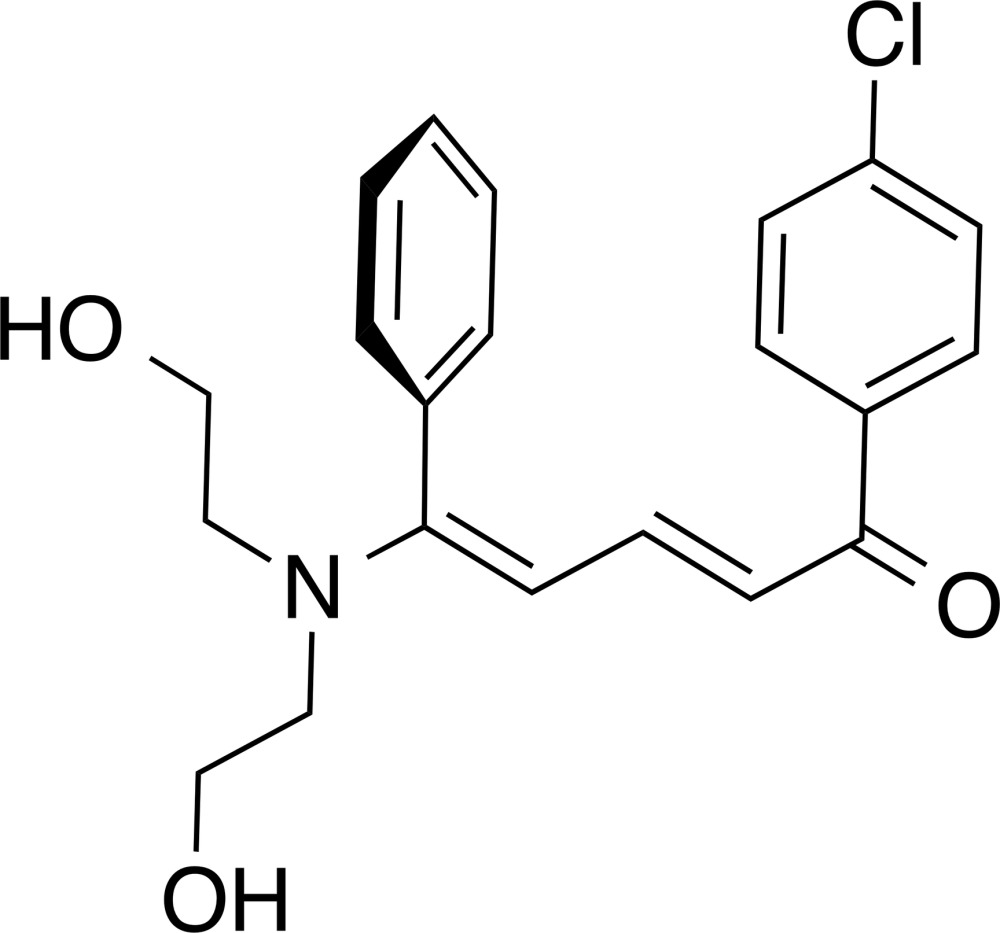


## Experimental   

### Crystal data  

C_21_H_22_ClNO_3_
*M* *_r_* = 371.85
Triclinic, 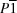

*a* = 6.6258 (1) Å
*b* = 11.0019 (2) Å
*c* = 13.8592 (3) Å
α = 110.980 (1)°
β = 99.401 (2)°
γ = 93.338 (1)°
*V* = 923.14 (3) Å^3^
*Z* = 2
Cu *K*α radiation
μ = 2.00 mm^−1^
*T* = 120 K
0.18 × 0.06 × 0.06 mm

### Data collection  

Bruker APEXII CCD diffractometer
Absorption correction: multi-scan (*SADABS*; Bruker, 2005 ▸) *T*_min_ = 0.715, *T*_max_ = 0.890
8297 measured reflections
3028 independent reflections
2686 reflections with *I* > 2σ(*I*)
*R*_int_ = 0.030

### Refinement  

*R*[*F* ^2^ > 2σ(*F* ^2^)] = 0.031
*wR*(*F* ^2^) = 0.092
*S* = 0.99
3028 reflections
235 parameters
H-atom parameters constrained
Δρ_max_ = 0.19 e Å^−3^
Δρ_min_ = −0.20 e Å^−3^

### 

Data collection: *APEX2* (Bruker, 2005 ▸); cell refinement: *SAINT* (Bruker, 2005 ▸); data reduction: *SAINT*; program(s) used to solve structure: *SHELXTL* (Sheldrick, 2008 ▸); program(s) used to refine structure: *SHELXTL*; molecular graphics: *SHELXTL*; software used to prepare material for publication: *SHELXTL* and *publCIF* (Westrip, 2010 ▸).

## Supplementary Material

Crystal structure: contains datablock(s) global, I. DOI: 10.1107/S2056989015019568/xu5877sup1.cif

Structure factors: contains datablock(s) I. DOI: 10.1107/S2056989015019568/xu5877Isup2.hkl

Supporting information file. DOI: 10.1107/S2056989015019568/xu5877Isup3.cml

**Fig. 1.**
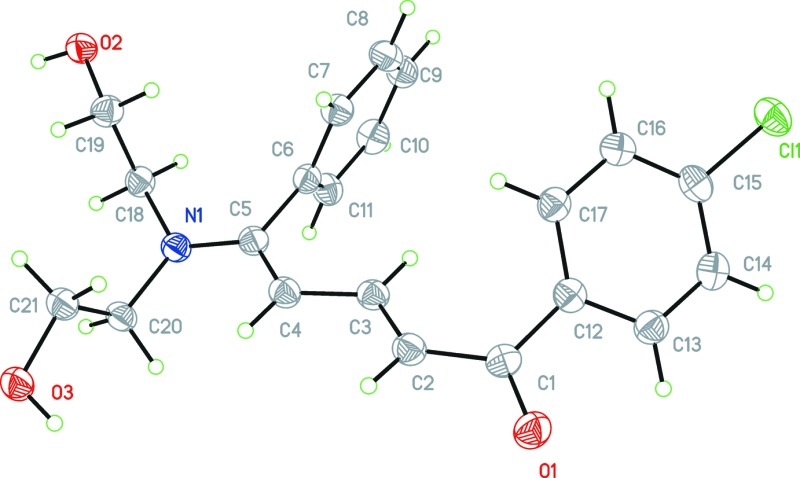
The molecular structure of the title compound. Displacement ellipsoids are drawn at the 50% probability level.

. DOI: 10.1107/S2056989015019568/xu5877fig1.tif
The mol­ecular structure of the title compound. Displacement ellipsoids are drawn at the 50% probability level.

**Fig. 2.**
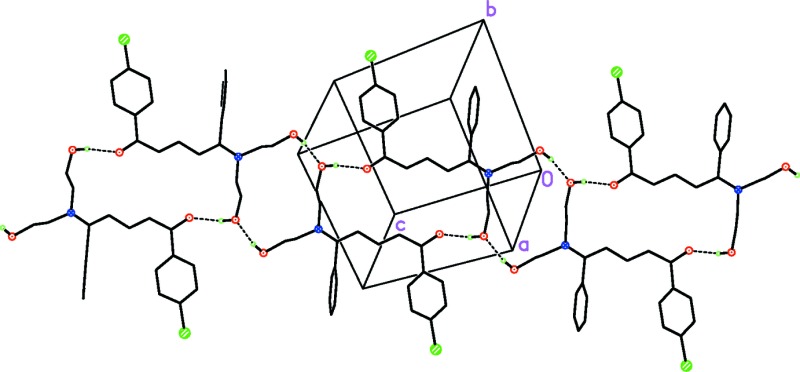
Fragment of a classic H-bonded chain (shown with dashed lines). The H(C) atoms are omitted for clarity.

. DOI: 10.1107/S2056989015019568/xu5877fig2.tif
Fragment of a classic H-bonded chain (shown with dashed lines). The H(C) atoms are omitted for clarity.

CCDC reference: 1431635

Additional supporting information:  crystallographic information; 3D view; checkCIF report

## Figures and Tables

**Table 1:** Hydrogen-bond geometry (, ) *Cg*1 is the centroid of the C12C17 ring.

*D*H*A*	*D*H	H*A*	*D* *A*	*D*H*A*
O2H2*B*O3^i^	0.84	1.92	2.7475(15)	168
O3H3*B*O1^ii^	0.84	1.87	2.6983(16)	169
C7H7*A*O1^iii^	0.95	2.59	3.497(2)	159
C20H20*B*O2^iv^	0.99	2.49	3.3396(18)	143
C21H21*A* *Cg*1^iii^	0.99	2.73	3.5791(17)	144

Symmetry codes: (i) 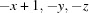; (ii) 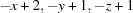; (iii) 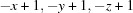; (iv) 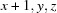.

## supplementary crystallographic information

### S1. Structural commentary

\ By the reaction between di­ethano­lamine and (*E*)-1-(4-chloro­phenyl)-5-phenyl­pent-2-en-4-yn-1-one the title compound was synthesized.

All bond lengths and valence angles are characteristic of single, double and aromatic bonds (Allen *et al.*, 1987), although the length of the C3—C4 bond (1.416 (2) Å) indicates slight delocalization of electron density along polyenone chain. In contrast with previously characterized 1-aryl-5-phenyl­pent-2,4-dien-1-ones (Kashino & Haisa, 1980; Fischer *et al.*, 2007a,b) Patil *et al.*, 2007; Zhao *et al.*, 2007; Silva *et al.*, 2011) and the (*E,Z*)-1-(4-chloro­phenyl)-5-phenyl-5-(phenyl­sulfanyl)penta-2,4-dien-1-\ one (Vologzhanina *et al.*, 2013), the title compound adopts the *cis*-orientation of C(3) and C(6) atoms in respect to the C(4)═C(5) do uble bond (Figure S1) which was previously observed only for (*E,E*)-1-(4-chloro­phenyl)-5-phenyl-5-(piperidin-1-yl)penta-2,4-dien-1-\ one (Golovanov *et al.*, 2014). Besides, it is the first representative of 1-aryl-5-phenyl­pent-2,4-dien-1-ones with the *s-trans* conformation of the enone fragment. As the result coplanarity between pentdienone and phenyl rings is absent, whilst the other 1,5-di­aryl­pentdienones are *quasi*-planar. The angles between the meanplane of the pent-2,4-dien-1-one chain (RMSD = 0.11 (8) Å) and those of chloro­phen-4-yl and phenyl rings are equal to, respectively, 137.65 (6) and 72.48 (4) °.

Due to the presence of two donor H(O) atoms, hydrogen bonding realizes in t he crystal of the title compound. Despite the presence of chlorine and nitr ogen atoms, only O—H···O bifurcate bonding was found with the oxygen atom of keto-group (Figure S2). The resulting H-bonded chain motif is characterized by O···O distances as short as 2.748 (2) and 2.698 (2) Å and OHO angles equal to 168 and 169 °.

### S2. Synthesis and crystallization

A solution of (499 mg, 1.87 mmol) (*E*)-1-(4-chloro­phenyl)-5-phenyl­pent-2-en-4-yn-1-one and (236 mg, 2.24 mmol) di­ethano­lamine in 95% EtOH (7 ml) was heated 10 h under reflux. The mixture was cooled, and the precipitate of adduct was filtered off, washed on a filter with 2 ml of cold 50% EtOH, and dried. Yield is 87 %. The single crystals of the product were obtained by slow crystallization from 95% EtOH. M.p. 370-371 K.

### S3. Refinement

H atoms were placed in the calculated positions with O—H = 0.84 and C—H = 0.95–0.99 Å, and refined in ride mode with U_iso_(H) = 1.5U_eq_(O) and 1.5U_eq_(C) for methyl H atoms and 1.2U_iso_(C) for the others.

### Crystal data

**Table:**

C_21_H_22_ClNO_3_	*Z* = 2
*M**_r_* = 371.85	*F*(000) = 392
Triclinic, *P*1	*D*_x_ = 1.338 Mg m^−^^3^
Hall symbol: -P 1	Melting point: 370 K
*a* = 6.6258 (1) Å	Cu *K*α radiation, λ = 1.54178 Å
*b* = 11.0019 (2) Å	Cell parameters from 2512 reflections
*c* = 13.8592 (3) Å	θ = 3.5–67.5°
α = 110.980 (1)°	µ = 2.00 mm^−^^1^
β = 99.401 (2)°	*T* = 120 K
γ = 93.338 (1)°	Needle, yellow
*V* = 923.14 (3) Å^3^	0.18 × 0.06 × 0.06 mm

### Data collection

**Table:**

Bruker APEXII CCD diffractometer	3028 independent reflections
Radiation source: fine-focus sealed tube	2686 reflections with *I* > 2σ(*I*)
Graphite monochromator	*R*_int_ = 0.030
φ and ω scans	θ_max_ = 64.9°, θ_min_ = 3.5°
Absorption correction: multi-scan (*SADABS*; Bruker, 2005)	*h* = −7→7
*T*_min_ = 0.715, *T*_max_ = 0.890	*k* = −12→12
8297 measured reflections	*l* = −14→16

### Refinement

**Table:**

Refinement on *F*^2^	Primary atom site location: structure-invariant direct methods
Least-squares matrix: full	Secondary atom site location: difference Fourier map
*R*[*F*^2^ > 2σ(*F*^2^)] = 0.031	Hydrogen site location: inferred from neighbouring sites
*wR*(*F*^2^) = 0.092	H-atom parameters constrained
*S* = 0.99	*w* = 1/[σ^2^(*F*_o_^2^) + (0.060*P*)^2^ + 0.130*P*] where *P* = (*F*_o_^2^ + 2*F*_c_^2^)/3
3028 reflections	(Δ/σ)_max_ = 0.005
235 parameters	Δρ_max_ = 0.19 e Å^−^^3^
0 restraints	Δρ_min_ = −0.20 e Å^−^^3^

### Special details

**Table:**

Geometry. All e.s.d.'s (except the e.s.d. in the dihedral angle between two l.s. planes) are estimated using the full covariance matrix. The cell e.s.d.'s are taken into account individually in the estimation of e.s.d.'s in distances, angles and torsion angles; correlations between e.s.d.'s in cell parameters are only used when they are defined by crystal symmetry. An approximate (isotropic) treatment of cell e.s.d.'s is used for estimating e.s.d.'s involving l.s. planes.
Refinement. Refinement of *F*^2^ against ALL reflections. The weighted *R*-factor *wR* and goodness of fit *S* are based on *F*^2^, conventional *R*-factors *R* are based on *F*, with *F* set to zero for negative *F*^2^. The threshold expression of *F*^2^ > σ(*F*^2^) is used only for calculating *R*-factors(gt) *etc*. and is not relevant to the choice of reflections for refinement. *R*-factors based on *F*^2^ are statistically about twice as large as those based on *F*, and *R*- factors based on ALL data will be even larger.

### Fractional atomic coordinates and isotropic or equivalent isotropic displacement parameters (Å^2^)

**Table:**

	*x*	*y*	*z*	*U*_iso_*/*U*_eq_	
Cl1	0.20029 (6)	1.12487 (4)	0.63194 (3)	0.03690 (14)	
O1	0.91844 (17)	0.73532 (11)	0.65905 (8)	0.0324 (3)	
O2	0.01218 (16)	0.13121 (10)	−0.03794 (8)	0.0299 (3)	
H2B	0.0429	0.0713	−0.0890	0.045*	
O3	0.83459 (16)	0.06098 (10)	0.18632 (8)	0.0307 (3)	
H3B	0.9239	0.1182	0.2330	0.046*	
N1	0.52198 (18)	0.30611 (12)	0.12564 (9)	0.0246 (3)	
C1	0.7721 (2)	0.71163 (14)	0.58302 (11)	0.0256 (3)	
C2	0.7492 (2)	0.59076 (14)	0.49358 (12)	0.0266 (3)	
H2A	0.8258	0.5243	0.5034	0.032*	
C3	0.6301 (2)	0.56068 (14)	0.39630 (12)	0.0249 (3)	
H3A	0.5444	0.6229	0.3857	0.030*	
C4	0.6263 (2)	0.44205 (14)	0.30992 (12)	0.0254 (3)	
H4A	0.7134	0.3803	0.3201	0.030*	
C5	0.5039 (2)	0.41119 (14)	0.21228 (12)	0.0241 (3)	
C6	0.3479 (2)	0.49831 (14)	0.19510 (11)	0.0246 (3)	
C7	0.1775 (2)	0.51077 (15)	0.24391 (12)	0.0274 (3)	
H7A	0.1595	0.4631	0.2877	0.033*	
C8	0.0346 (2)	0.59274 (16)	0.22843 (13)	0.0318 (3)	
H8A	−0.0810	0.6011	0.2618	0.038*	
C9	0.0598 (2)	0.66242 (15)	0.16460 (13)	0.0322 (4)	
H9A	−0.0384	0.7182	0.1540	0.039*	
C10	0.2289 (3)	0.65052 (15)	0.11611 (13)	0.0325 (4)	
H10A	0.2460	0.6980	0.0720	0.039*	
C11	0.3729 (2)	0.56964 (15)	0.13177 (12)	0.0295 (3)	
H11A	0.4894	0.5628	0.0991	0.035*	
C12	0.6260 (2)	0.81178 (15)	0.58804 (11)	0.0254 (3)	
C13	0.7033 (2)	0.94446 (15)	0.64078 (12)	0.0290 (3)	
H13A	0.8468	0.9689	0.6691	0.035*	
C14	0.5731 (3)	1.04078 (15)	0.65230 (12)	0.0311 (3)	
H14A	0.6268	1.1309	0.6867	0.037*	
C15	0.3643 (2)	1.00379 (15)	0.61308 (12)	0.0296 (3)	
C16	0.2823 (2)	0.87320 (15)	0.56061 (12)	0.0285 (3)	
H16A	0.1383	0.8495	0.5337	0.034*	
C17	0.4143 (2)	0.77749 (15)	0.54807 (11)	0.0267 (3)	
H17A	0.3600	0.6877	0.5119	0.032*	
C18	0.3543 (2)	0.25212 (14)	0.03273 (11)	0.0262 (3)	
H18A	0.4102	0.1966	−0.0281	0.031*	
H18B	0.2993	0.3252	0.0155	0.031*	
C19	0.1793 (2)	0.17064 (15)	0.04944 (12)	0.0287 (3)	
H19A	0.2299	0.0920	0.0590	0.034*	
H19B	0.1316	0.2230	0.1142	0.034*	
C20	0.6923 (2)	0.22827 (14)	0.13012 (12)	0.0263 (3)	
H20A	0.8160	0.2875	0.1771	0.032*	
H20B	0.7240	0.1899	0.0587	0.032*	
C21	0.6511 (2)	0.11742 (15)	0.16928 (12)	0.0277 (3)	
H21A	0.5997	0.1523	0.2359	0.033*	
H21B	0.5436	0.0492	0.1165	0.033*	

### Atomic displacement parameters (Å^2^)

**Table:**

	*U*^11^	*U*^22^	*U*^33^	*U*^12^	*U*^13^	*U*^23^
Cl1	0.0381 (2)	0.0326 (2)	0.0451 (3)	0.01395 (16)	0.01386 (17)	0.01676 (17)
O1	0.0319 (6)	0.0311 (6)	0.0294 (6)	0.0036 (4)	−0.0017 (4)	0.0091 (4)
O2	0.0251 (5)	0.0301 (6)	0.0299 (6)	0.0051 (4)	0.0018 (4)	0.0072 (4)
O3	0.0286 (6)	0.0287 (5)	0.0301 (6)	0.0081 (4)	0.0001 (4)	0.0073 (4)
N1	0.0233 (6)	0.0258 (6)	0.0246 (7)	0.0044 (5)	0.0055 (5)	0.0087 (5)
C1	0.0249 (7)	0.0279 (7)	0.0256 (8)	0.0010 (6)	0.0049 (6)	0.0122 (6)
C2	0.0246 (7)	0.0262 (7)	0.0300 (8)	0.0047 (6)	0.0052 (6)	0.0116 (6)
C3	0.0227 (7)	0.0255 (7)	0.0286 (8)	0.0029 (5)	0.0069 (6)	0.0117 (6)
C4	0.0248 (7)	0.0251 (7)	0.0283 (8)	0.0049 (6)	0.0064 (6)	0.0116 (6)
C5	0.0230 (7)	0.0236 (7)	0.0277 (8)	0.0019 (5)	0.0081 (5)	0.0106 (6)
C6	0.0248 (7)	0.0227 (7)	0.0236 (8)	0.0023 (6)	0.0026 (5)	0.0066 (5)
C7	0.0277 (8)	0.0278 (7)	0.0290 (8)	0.0031 (6)	0.0068 (6)	0.0128 (6)
C8	0.0267 (8)	0.0323 (8)	0.0377 (9)	0.0067 (6)	0.0090 (6)	0.0129 (6)
C9	0.0314 (8)	0.0294 (8)	0.0348 (9)	0.0083 (6)	0.0008 (6)	0.0125 (6)
C10	0.0392 (9)	0.0305 (8)	0.0310 (9)	0.0042 (7)	0.0044 (6)	0.0165 (6)
C11	0.0325 (8)	0.0290 (8)	0.0287 (8)	0.0055 (6)	0.0092 (6)	0.0113 (6)
C12	0.0283 (8)	0.0286 (7)	0.0211 (8)	0.0042 (6)	0.0066 (5)	0.0105 (6)
C13	0.0278 (8)	0.0305 (8)	0.0274 (8)	0.0024 (6)	0.0046 (6)	0.0099 (6)
C14	0.0364 (9)	0.0263 (7)	0.0290 (8)	0.0033 (6)	0.0061 (6)	0.0088 (6)
C15	0.0343 (8)	0.0310 (8)	0.0288 (8)	0.0109 (6)	0.0116 (6)	0.0142 (6)
C16	0.0272 (8)	0.0324 (8)	0.0275 (8)	0.0043 (6)	0.0057 (6)	0.0130 (6)
C17	0.0289 (8)	0.0279 (7)	0.0231 (8)	0.0021 (6)	0.0055 (6)	0.0092 (6)
C18	0.0276 (8)	0.0270 (7)	0.0227 (8)	0.0040 (6)	0.0044 (6)	0.0077 (6)
C19	0.0277 (8)	0.0301 (8)	0.0273 (8)	0.0031 (6)	0.0039 (6)	0.0105 (6)
C20	0.0230 (7)	0.0284 (7)	0.0271 (8)	0.0069 (6)	0.0072 (6)	0.0084 (6)
C21	0.0258 (8)	0.0277 (7)	0.0283 (8)	0.0062 (6)	0.0041 (6)	0.0089 (6)

### Geometric parameters (Å, º)

**Table:**

Cl1—C15	1.7423 (15)	C9—H9A	0.9500
O1—C1	1.2483 (19)	C10—C11	1.385 (2)
O2—C19	1.4179 (18)	C10—H10A	0.9500
O2—H2B	0.8400	C11—H11A	0.9500
O3—C21	1.4209 (17)	C12—C17	1.397 (2)
O3—H3B	0.8400	C12—C13	1.398 (2)
N1—C5	1.3644 (19)	C13—C14	1.387 (2)
N1—C20	1.4624 (18)	C13—H13A	0.9500
N1—C18	1.4672 (19)	C14—C15	1.380 (2)
C1—C2	1.436 (2)	C14—H14A	0.9500
C1—C12	1.499 (2)	C15—C16	1.386 (2)
C2—C3	1.361 (2)	C16—C17	1.389 (2)
C2—H2A	0.9500	C16—H16A	0.9500
C3—C4	1.416 (2)	C17—H17A	0.9500
C3—H3A	0.9500	C18—C19	1.522 (2)
C4—C5	1.373 (2)	C18—H18A	0.9900
C4—H4A	0.9500	C18—H18B	0.9900
C5—C6	1.4978 (19)	C19—H19A	0.9900
C6—C11	1.393 (2)	C19—H19B	0.9900
C6—C7	1.397 (2)	C20—C21	1.530 (2)
C7—C8	1.388 (2)	C20—H20A	0.9900
C7—H7A	0.9500	C20—H20B	0.9900
C8—C9	1.385 (2)	C21—H21A	0.9900
C8—H8A	0.9500	C21—H21B	0.9900
C9—C10	1.387 (2)		
			
C19—O2—H2B	109.5	C13—C12—C1	118.42 (13)
C21—O3—H3B	109.5	C14—C13—C12	120.79 (15)
C5—N1—C20	120.41 (12)	C14—C13—H13A	119.6
C5—N1—C18	121.58 (12)	C12—C13—H13A	119.6
C20—N1—C18	117.00 (11)	C15—C14—C13	119.04 (15)
O1—C1—C2	119.50 (13)	C15—C14—H14A	120.5
O1—C1—C12	118.48 (13)	C13—C14—H14A	120.5
C2—C1—C12	122.02 (13)	C14—C15—C16	121.69 (14)
C3—C2—C1	127.31 (13)	C14—C15—Cl1	118.85 (12)
C3—C2—H2A	116.3	C16—C15—Cl1	119.45 (12)
C1—C2—H2A	116.3	C17—C16—C15	118.86 (14)
C2—C3—C4	123.85 (13)	C17—C16—H16A	120.6
C2—C3—H3A	118.1	C15—C16—H16A	120.6
C4—C3—H3A	118.1	C16—C17—C12	120.77 (14)
C5—C4—C3	123.74 (13)	C16—C17—H17A	119.6
C5—C4—H4A	118.1	C12—C17—H17A	119.6
C3—C4—H4A	118.1	N1—C18—C19	112.61 (12)
N1—C5—C4	123.35 (13)	N1—C18—H18A	109.1
N1—C5—C6	116.34 (13)	C19—C18—H18A	109.1
C4—C5—C6	120.23 (13)	N1—C18—H18B	109.1
C11—C6—C7	119.24 (13)	C19—C18—H18B	109.1
C11—C6—C5	120.35 (13)	H18A—C18—H18B	107.8
C7—C6—C5	120.40 (13)	O2—C19—C18	110.76 (12)
C8—C7—C6	120.05 (14)	O2—C19—H19A	109.5
C8—C7—H7A	120.0	C18—C19—H19A	109.5
C6—C7—H7A	120.0	O2—C19—H19B	109.5
C9—C8—C7	120.30 (15)	C18—C19—H19B	109.5
C9—C8—H8A	119.9	H19A—C19—H19B	108.1
C7—C8—H8A	119.9	N1—C20—C21	114.63 (12)
C10—C9—C8	119.85 (14)	N1—C20—H20A	108.6
C10—C9—H9A	120.1	C21—C20—H20A	108.6
C8—C9—H9A	120.1	N1—C20—H20B	108.6
C11—C10—C9	120.18 (15)	C21—C20—H20B	108.6
C11—C10—H10A	119.9	H20A—C20—H20B	107.6
C9—C10—H10A	119.9	O3—C21—C20	110.36 (12)
C10—C11—C6	120.38 (14)	O3—C21—H21A	109.6
C10—C11—H11A	119.8	C20—C21—H21A	109.6
C6—C11—H11A	119.8	O3—C21—H21B	109.6
C17—C12—C13	118.83 (14)	C20—C21—H21B	109.6
C17—C12—C1	122.62 (13)	H21A—C21—H21B	108.1
			
O1—C1—C2—C3	−164.68 (15)	C5—C6—C11—C10	−179.81 (14)
C12—C1—C2—C3	14.9 (2)	O1—C1—C12—C17	−142.76 (15)
C1—C2—C3—C4	175.65 (14)	C2—C1—C12—C17	37.7 (2)
C2—C3—C4—C5	179.10 (15)	O1—C1—C12—C13	33.1 (2)
C20—N1—C5—C4	−7.3 (2)	C2—C1—C12—C13	−146.49 (15)
C18—N1—C5—C4	160.89 (14)	C17—C12—C13—C14	−0.8 (2)
C20—N1—C5—C6	169.64 (12)	C1—C12—C13—C14	−176.78 (13)
C18—N1—C5—C6	−22.20 (19)	C12—C13—C14—C15	1.6 (2)
C3—C4—C5—N1	170.29 (14)	C13—C14—C15—C16	−1.4 (2)
C3—C4—C5—C6	−6.5 (2)	C13—C14—C15—Cl1	177.71 (11)
N1—C5—C6—C11	−64.96 (19)	C14—C15—C16—C17	0.5 (2)
C4—C5—C6—C11	112.05 (17)	Cl1—C15—C16—C17	−178.65 (11)
N1—C5—C6—C7	116.19 (15)	C15—C16—C17—C12	0.4 (2)
C4—C5—C6—C7	−66.80 (19)	C13—C12—C17—C16	−0.2 (2)
C11—C6—C7—C8	0.5 (2)	C1—C12—C17—C16	175.64 (13)
C5—C6—C7—C8	179.36 (14)	C5—N1—C18—C19	−76.12 (17)
C6—C7—C8—C9	0.1 (2)	C20—N1—C18—C19	92.42 (15)
C7—C8—C9—C10	−0.2 (2)	N1—C18—C19—O2	174.26 (11)
C8—C9—C10—C11	−0.3 (2)	C5—N1—C20—C21	86.90 (16)
C9—C10—C11—C6	0.9 (2)	C18—N1—C20—C21	−81.78 (16)
C7—C6—C11—C10	−0.9 (2)	N1—C20—C21—O3	−171.02 (11)

### Hydrogen-bond geometry (Å, º)

Cg1 is the centroid of the C12–C17 ring.

**Table:**

*D*—H···*A*	*D*—H	H···*A*	*D*···*A*	*D*—H···*A*
O2—H2*B*···O3^i^	0.84	1.92	2.7475 (15)	168
O3—H3*B*···O1^ii^	0.84	1.87	2.6983 (16)	169
C7—H7*A*···O1^iii^	0.95	2.59	3.497 (2)	159
C20—H20*B*···O2^iv^	0.99	2.49	3.3396 (18)	143
C21—H21*A*···*Cg*1^iii^	0.99	2.73	3.5791 (17)	144

Symmetry codes: (i) −*x*+1, −*y*, −*z*; (ii) −*x*+2, −*y*+1, −*z*+1; (iii) −*x*+1, −*y*+1, −*z*+1; (iv) *x*+1, *y*, *z*.
