# Supplementary figures and images for: Crystal structure of (2E,4E)-5-[bis­(2-hy­droxy­eth­yl)amino]-1-(4-chloro­phen­yl)-5-phenyl­penta-2,4-dien-1-one
Source: Acta Crystallogr E Crystallogr Commun. 2015 Oct 24;71(Pt 11):o870–1. doi: 10.1107/S2056989015019568 (PMC4645000; doi:10.1107/S2056989015019568)

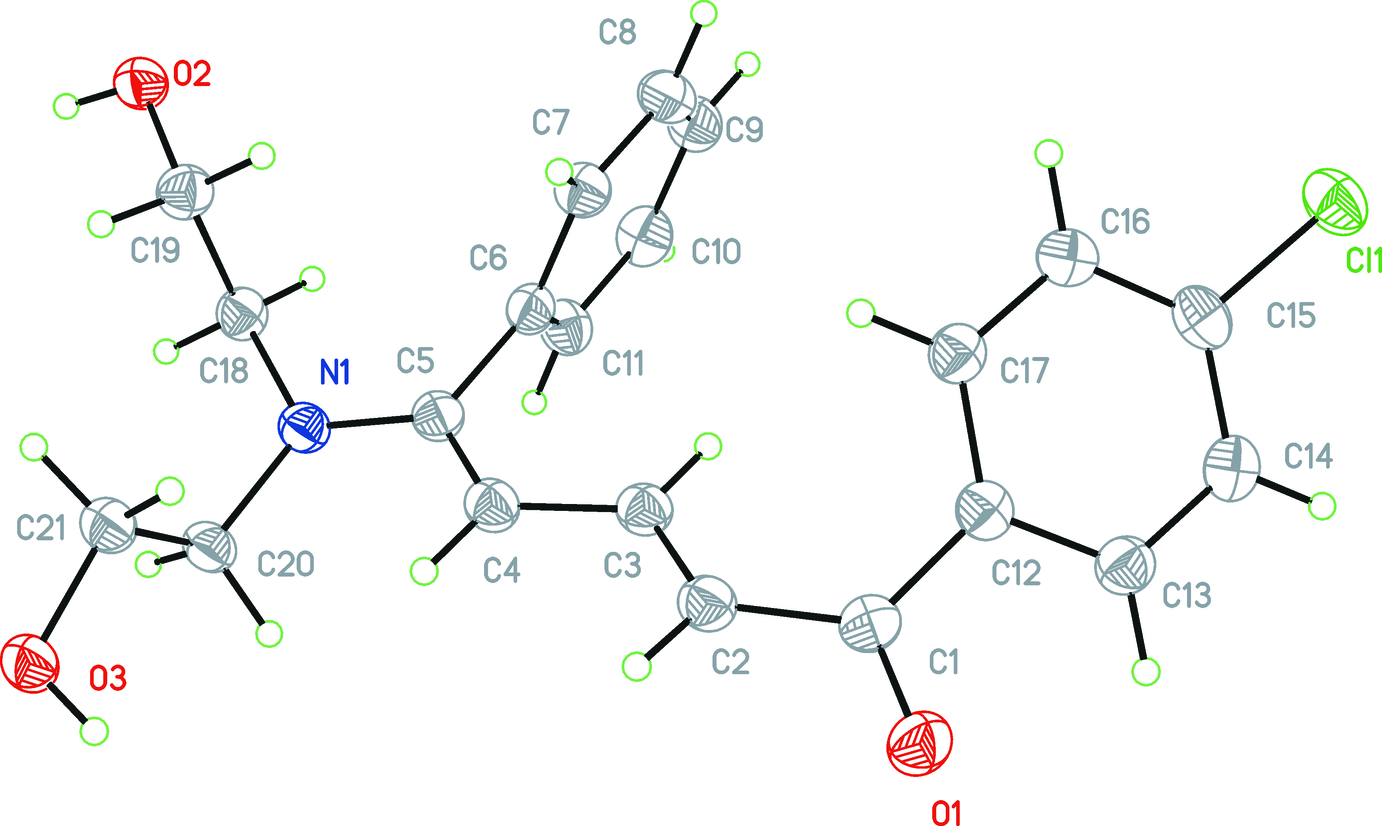

Supplement: Supplementary file 4 [file e-71-0o870-fig1.tif]

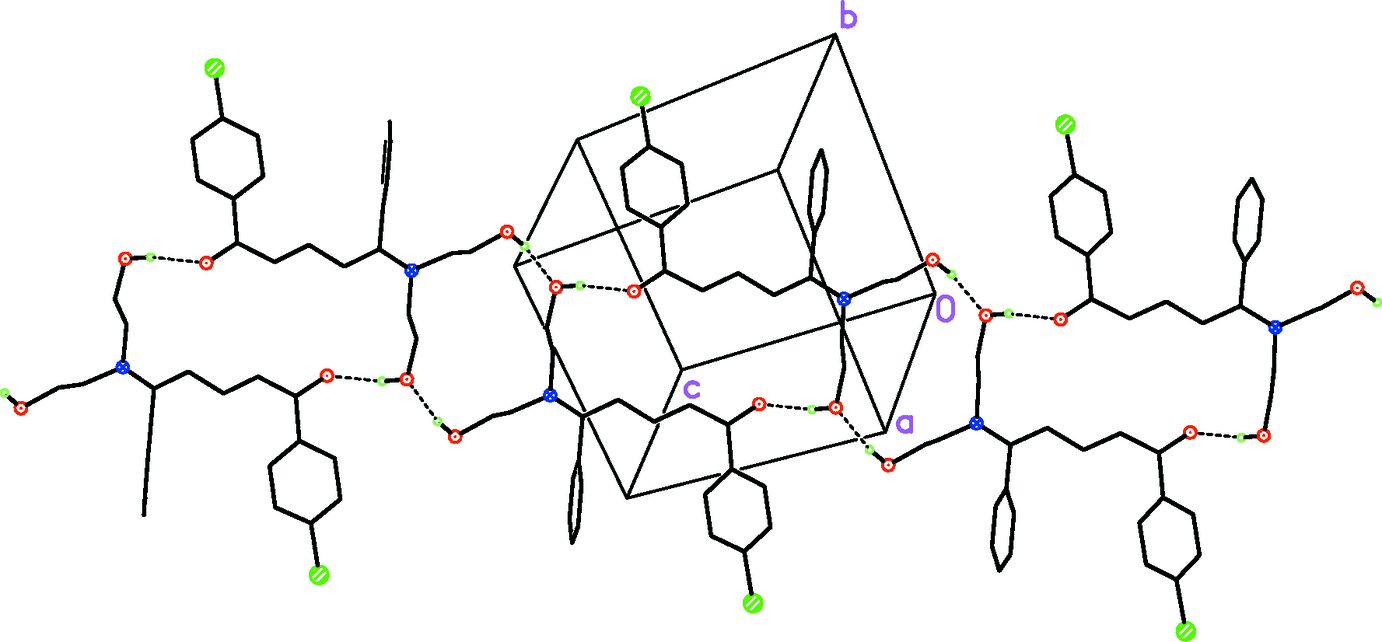

Supplement: Supplementary file 5 [file e-71-0o870-fig2.tif]
